# Mental Health, Burnout and Problematic Drinking in Norwegian Medical Doctors

**DOI:** 10.1192/j.eurpsy.2022.88

**Published:** 2022-09-01

**Authors:** R. Tyssen

**Affiliations:** Institute of Basic Medical Sciences, Faculty of Medicine, University of Oslo, Department Of Behavioural Medicine, Oslo, Norway

**Keywords:** mental health, alcohol, Stress, physicians

## Abstract

Previous studies have found relatively good physical health in doctors, whereas several studies now report relatively high levels of stress and burnout among them. With the exception of higher suicide rates, we have less evidence of poorer mental health among doctors than among other professionals. The elevated suicide rate may represent the tip of an iceberg of frustration and inadequate mental health care among medical doctors. There are very few longitudinal studies that can identify possible risk factors and causality. The Longitudinal Study of Norwegian Medical Students and Doctors (NORDOC) has since 1993/94 followed repeatedly two cohorts of medical students (N=1052) in seven waves during 25 years (Facebook: @docsinrush). Outcomes presented here are on mental health, burnout and problematic drinking. There are two main hypotheses with regard to possible risk factors. First, it may be due to individual factors such as personality traits, past mental health problems etc. Second, contextual stress may influence mental health among doctors, whether this be unhealthy working conditions or negative life events (i.e. stress outside of work). The presentation will give and overview of both individual and work-related predictors of stress and mental health problems among Norwegian physicians. Individual and organizational interventions to reduce stress and physician burnout will also be dealt with.

**Disclosure:**

No significant relationships.

